# Real-time interactive digital healthcare system for post-operative breast cancer patients: study protocol for a randomized controlled trial

**DOI:** 10.1186/s13063-021-05535-8

**Published:** 2021-08-19

**Authors:** Hae-Yeon Park, Kyung Eun Nam, Jae-Young Lim, Seung Mi Yeo, Jong In Lee, Ji Hye Hwang

**Affiliations:** 1 Department of Rehabilitation Medicine, Seoul St. Mary’s Hospital, College of Medicine, The Catholic University of Korea, 222.Banpo-dearo, Seocho-gu, Seoul, Republic of Korea; 2 Department of Rehabilitation Medicine, Seoul National University Bundang Hospital, Seongnam, Gyeonggi-Do Republic of Korea; 3 Department of Physical and Rehabilitation Medicine, Samsung Medical Center, Sungkyunkwan University School of Medicine, 81 Irwon-Ro Gangnam-gu, Seoul, Republic of Korea

**Keywords:** Augmented reality, Breast cancer, Rehabilitation

## Abstract

**Background:**

After receiving breast cancer surgery or reconstruction, shoulder dysfunctions including weakness, post-operative pain, shoulder joint instability, and limited range of motion (ROM) often occur. Due to limited ROM, patients may suffer difficulty in activities of daily living, and quality of life may be reduced. The objective of this study is to compare the effects on shoulder ROM of a real-time interactive digital healthcare system and brochure-based home rehabilitation program in post-operative breast cancer patients.

**Methods:**

This study is a prospective, multi-center, assessor-blinded randomized controlled trial. The study aims to recruit 100 breast cancer patients exhibiting limited shoulder ROM after undergoing axillary lymph node dissection or breast reconstruction following mastectomy. Patients will be randomly assigned to two groups for 8 weeks of rehabilitation: a digital healthcare system rehabilitation (intervention) group and a brochure-based rehabilitation (control) group. The primary outcome is the change in ROM of the affected shoulder between baseline and 12 weeks after enrollment. Secondary outcomes include pain in the affected shoulder, as measured using a numerical rating scale, functional outcomes (QuickDASH scores), and quality of life (FACT-B and EQ-5D-5L scores), all of which will be measured on enrollment and 4, 8, and 12 weeks thereafter.

**Discussion:**

This study will compare the effectiveness of a newly developed, augmented reality-based real-time interactive digital healthcare system with that of brochure-based home rehabilitation for improving the shoulder ROM, pain, functional outcomes, and quality of life of post-operative breast cancer patients.

**Trial registration:**

ClinicalTrials.govNCT04316156. Registered on 20 March 2020.

**Supplementary Information:**

The online version contains supplementary material available at 10.1186/s13063-021-05535-8.

## Background

Breast cancer is one of the most common forms of cancer, and cancer death, among females; it accounted for 11.6% of the 18.1 million new cancer cases worldwide in 2018 [1]. Shoulder disabilities frequently occur following breast cancer surgery, especially after axillary lymph node dissection (ALND) and mastectomy [2–6]. Weakness, lymphedema, limited range of motion (ROM), and pain in the upper extremity on the operated side are common symptoms [3, 4]. After mastectomy, several reconstruction methods are commonly considered, such as expander/implant-based reconstruction and autologous reconstruction using abdominal tissue or latissimus dorsi muscle. Decreased strength of the affected upper limb, reduced shoulder joint stability and shoulder ROM, and functional limitations may arise after breast cancer reconstruction [7, 8]. Due to these postsurgical complications, which are also associated with emotional changes, a high proportion of patients complain of decreased quality of life and difficulty performing daily activities [4, 8–10].

Exercise is reportedly effective for improving shoulder dysfunction and quality of life after breast cancer surgery. After ALND, participants who received physical therapy and posture education showed improvement in postsurgical shoulder pain and the ability to perform daily activities [9]. Shoulder function and health-related quality of life also significantly improved after upper limb exercises and muscle relaxation training [11]. Furthermore, early rehabilitation, including shoulder ROM and strengthening exercises, improved shoulder ROM after breast cancer surgery [12].

Recently, new technologies facilitating rehabilitation, such as virtual reality (VR) and augmented reality (AR), have been introduced. One study examined the effectiveness of VR-based Xbox Kinect (Microsoft Corp., Redmond, WA, USA) games for rehabilitating breast cancer patients after surgery [13]. To our knowledge, however, outcomes of real-time interactive AR-based rehabilitation programs for breast cancer surgery patients have not yet been reported. Therefore, we aimed to investigate the usefulness of AR system for shoulder dysfunction in post-operative breast cancer patients.

**Fig. 1:**
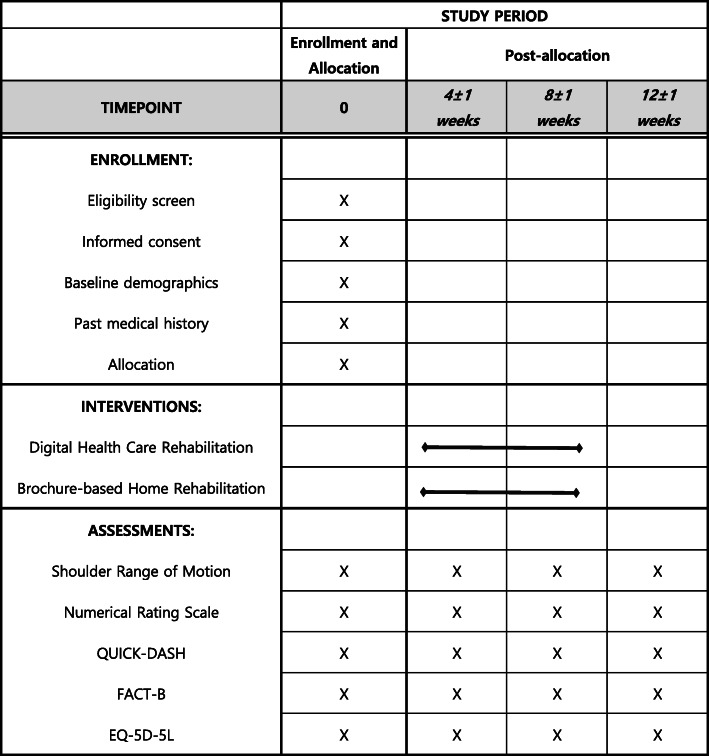
The schedule of enrollment, interventions, and assessments

The primary objective of this prospective randomized controlled study is to compare the effectiveness of an AR-based digital healthcare system using motion sensors with that of brochure-based home rehabilitation for shoulder ROM after breast cancer surgery. The study protocol is reported according to the Standard Protocol Items: Recommendations for Interventional Trials (SPIRIT) guidelines (Additional file 1); a schematic is provided in Fig. 1.

## Methods/design

### Study design and setting

**Fig. 2:**
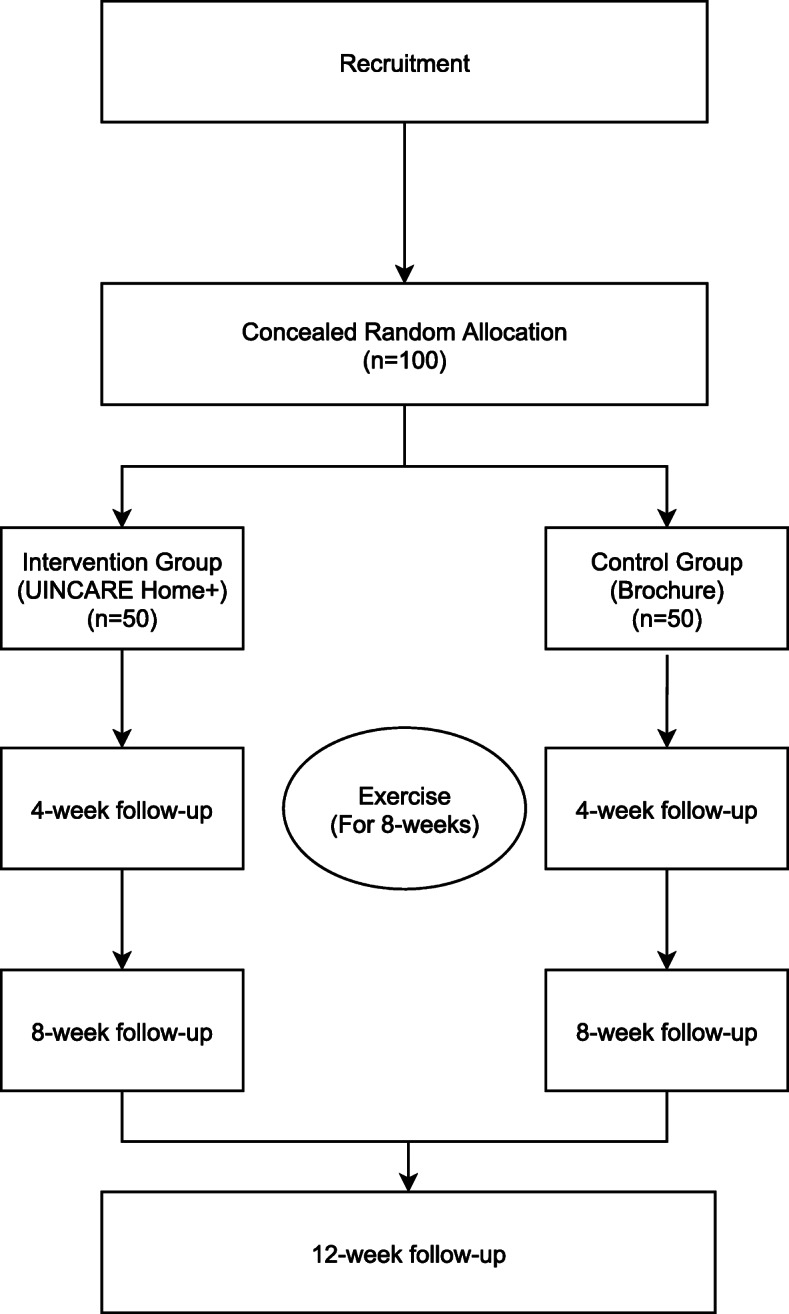
Flowchart of the study

This is a prospective, multi-center, assessor-blinded randomized controlled study. Breast cancer patients who underwent breast cancer surgery in two university hospitals in South Korea (Samsung Medical Center, Sungkyunkwan University School of Medicine and Seoul St. Mary’s Hospital, College of Medicine, The Catholic University of Korea) will be recruited. The enrolled participants will be randomly allocated to either the digital healthcare rehabilitation group or the brochure-based rehabilitation group. Both groups will receive rehabilitation for 8 weeks. A flowchart of the study design is shown in Fig. 2.

### Eligibility criteria

The inclusion criteria for post-operative breast cancer patients are as follows: (a) aged between 20 and 70 years, (b) underwent ALND or breast reconstruction following mastectomy, (c) ≤ 8 weeks since the operation, and (d) limited ROM of the affected shoulder (flexion or abduction < 160°). The exclusion criteria are as follows: (a) history of bilateral breast cancer surgery, (b) existence of shoulder pain and limited ROM before the surgery, (c) communication difficulty that interferes with following the exercise instruction, and (d) general deconditioning that enables daily exercise. All enrolled participants, regardless of the groups, are allowed to receive pre-scheduled chemotherapy, radiotherapy, and hormone therapy during the study. In case of participant’s willing to withdrawal, the participants will be dropped out. After the clinical trial, patients will continue pre-scheduled treatments for breast cancer and physical therapy will be prescribed if they want rehabilitation therapy in our hospital, regardless of being dropped out or completing the trial.

The study was approved by our institutional review board (approval number: KC20ENDI0154, SMC-2019-05-021), and written informed consent will be obtained from all participants. Any modification to the protocol will be reported to institutional review board. Institutional review board will also monitor the clinical trial process and any complications occurred during the study.

### Randomization, allocation concealment, and blinding

Participants meeting the eligibility criteria will be randomly assigned to either the digital healthcare or brochure-based rehabilitation group, in a 1:1 ratio using a block randomization procedure through computer-generated sequences with block size of four. One research team member will independently allocate the participants to groups using the sequences generated by computer. The researchers involved in the randomization process will not participate in the enrollment and assessment processes, and the allocation will be concealed by sealed notes. Therefore, both the participants and researchers involved in enrollment and assessment will be blinded to the allocation process. An experienced physical or occupational therapist blinded to the group allocations will assess the primary outcome.

### Intervention

#### Digital healthcare system (intervention) group

In the intervention group, rehabilitation will be provided via the UINCARE Home+ rehabilitation system (UINCARE Corp., Seoul, Korea), which will be delivered to the participants after enrollment. The UINCARE Home+ is based on infrared technology and includes an Xbox One Kinect for Windows® (Microsoft Corp.) motion capture device. It provides real-time feedback directly to the user by tracking the movements of 25 joints, in both the upper and lower extremities, in three-dimensional space. Physicians can prescribe exercise to the user through the Internet. Task performance, including repetitions and movement accuracy, is recorded in real time.

After logging in to the UINCARE Home+ system, participants can perform daily exercises, as prescribed by the physician. The exercise program consists of two components, with two levels each (total of four levels). After performing the exercises of a given level for 2 weeks, the participant progresses to the next level. At a follow-up assessment 4 weeks after enrollment, the program may be recalibrated by the physician. If the participant experiences difficulty performing the exercises at one level, that level will be maintained for a further 2 weeks. The entire exercise program is shown in Table 1. Each session comprises 12–14 workouts, with a 2.5-min warm-up period and 45-s cool-down period. The total session length is approximately 30 min. Each workout is repeated five times, and participants will be asked to perform an exercise session more than once a day on every day of the week. The exercise program will be performed over 8 weeks.

**Table 1:** The entire exercise program

	Part 1 (level 1)	Part 1 (level 2)	Part 2 (level 3)	Part 2 (Level 4)
Warm up	Deep breathing Trunk twist (Rt./Lt.)	Deep breathing Trunk twist (Rt./Lt.)	Deep breathing Trunk twist (Rt./Lt.)	Deep breathing Trunk twist (Rt./Lt.)
Main workout	Passive forward flexion 90° Passive external rotation 45° Passive abduction 90° Trunk rotation with both shoulder forward flexion 90° (Rt./Lt.) Active forward flexion 90° Active abduction 90° (both arm) Passive forward flexion 90° Passive external rotation 45° Passive abduction 90° Trunk rotation with both shoulder forward flexion 90° (Rt./Lt.) Active forward flexion 90° Active abduction 90° (both arm)	Passive forward flexion 180° Passive external rotation 90° Passive abduction 180° Active forward flexion 180° Active abduction 180° Active external rotation Pectoralis stretching^a^ Passive forward flexion 180° Passive external rotation 90° Passive abduction 180° Active forward flexion 180° Active abduction 180° Active external rotation Pectoralis stretching^a^	Passive forward flexion 180° Passive external rotation 90° Passive abduction 180° Forward flexion 90° with dumbbell Abduction 90° with dumbbell Pectoralis stretching^b^ Active forward flexion 180° Active abduction 180° Active external rotation 90° Forward flexion 90° with dumbbell Abduction 90° with dumbbell Pectoralis stretching^b^	Active forward flexion 180° Active abduction 180° Active external rotation 90° Forward flexion 180° with dumbbell Abduction 180° with dumbbell External rotation with dumbbell Biceps strengthening with dumbbell Pectoralis stretching^c^ Forward flexion 180° with dumbbell Abduction 180° with dumbbell External rotation with dumbbell Pectoralis stretching^c^
Cool down	Deep breathing	Deep breathing	Deep breathing	Deep breathing

^a^Pectoralis stretching is performed with both arms abducted

^b^Pectoralis stretching is performed with interlocking fingers behind the back and raising both arms up

^c^Pectoralis stretching is performed with interlocking fingers behind the occiput and moving both elbows backward

#### Brochure-based home rehabilitation (control) group

Participants allocated to the control group will receive a brochure detailing the same exercise program followed by the intervention group (Table 1). The brochure contains pictures and brief written summaries of each motion. Similar to the intervention group, the brochure-based home rehabilitation group will be assessed at a 4-week follow-up, and the level of exercise will be set according to the results of that assessment. The control group will also be asked to perform exercise sessions more than once a day on every day of the week. Adherence to the exercise program will be monitored using exercise log books, which will be provided at enrollment and the 4-week follow-up.

### Outcome measures

The primary outcome of the study is the change in ROM (flexion or abduction) in the affected shoulder between baseline and 12 weeks. Flexion, abduction, external rotation, and internal rotation will be measured both passively and actively in a sitting position. External and internal rotation will be measured with the shoulder abducted to 90° or as much as possible. ROM will be measured using a goniometer. Measurements will be performed on enrollment, and 4, 8, and 12 weeks thereafter, by a physical or occupational therapist blinded to the group assignments.

The secondary outcomes of this study are pain, functional outcomes, and quality of life. The intensity of pain in the affected shoulder will be measured using a numerical rating scale (NRS), which is a simple and commonly used method for rating pain intensity from 0 (*no pain*) to 10 (*worst pain imaginable*) [14]. Both maximum pain and general pain will be measured at each visit: maximum pain refers to the most intense pain experienced during the week before the visit, and general pain represents the average pain intensity during that week. Functional outcomes will be measured using the short-form version of the Disabilities of the Arm, Shoulder and Hand questionnaire (QuickDASH), a widely used self-report measure of upper extremity disability [15]. Higher QuickDASH scores reflect poorer functional outcomes. Quality of life will be measured using the Functional Assessment of Cancer Therapy-Breast (FACT-B) and EuroQol Five-dimensional (EQ-5D-5L) instruments. The FACT-B scale consists of 37 items and is specifically aimed at breast cancer patients. The four subdomains of FACT-B are physical, social/family, emotional, and functional well-being. Higher FACT-B scores reflect better the quality of life [16–18]. The EQ-5D-5L questionnaire captures health status based on five dimensions: mobility, self-care, usual activities, pain/discomfort, and anxiety/depression [19]. Each dimension is scored from 1 to 5, and health status is summarized as a five-digit code: “11111” represents no problems in any of the five dimensions, and “55555” represents the worst possible health status. Higher EQ-5D-5L scores indicate better health status [20]. Secondary outcomes will also be assessed, on enrollment and 4, 8, and 12 weeks thereafter. Time schedule of enrollment, interventions, assessments are presented in Fig. 1.

### Statistical analysis

#### Sample size

The standard deviation of ROM in the affected shoulder is expected to be 23°, and the difference in ROM between groups is expected to be 15° based on a previous study [9] and our own unpublished preliminary study. Using two-sample *Z* tests, and assuming homogeneity of variance, the sample size was estimated at 37 for each group with an alpha level of 5% and power of 80%. With an expected dropout rates of 20%, at least 47 participants are needed in each group, so enrollment of 100 participants (*n* = 50 per group) is planned.

#### Data management

Data related to the trial will be anonymized and recorded in password-protected case report forms (CRFs). Only authorized researchers will have access to the CRFs. No interim analysis will be performed prior to the end of the study. The collected data will be kept for 5 years after the end of the study and will be destroyed after that.

#### Data analysis

All statistical analyses will be performed using SPSS® Statistics (version 24.0; IBM Corp., Armonk, NY, USA). The Shapiro–Wilk test will be used to evaluate the normality of the data. The primary outcome will be analyzed using the independent *t* test or Mann–Whitney test, depending on the normality of the data. The secondary outcomes will be analyzed using a linear mixed model or generalized estimating equation. *P* values < 0.05 will be considered statistically significant. Both intention to treat and per protocol analyses will be performed.

## Discussion

Limitations of shoulder ROM after breast cancer surgery are common, and physical therapy is known to be effective [2–6, 12]. Patients usually receive physical therapy by attending the rehabilitation center. However, it is difficult for those living in remote areas to attend the center. In that case, brochure-based physical therapy could be considered, though feedback on exercise is not possible. With the advance of the technology, there have been efforts to apply VR and AR system in exercise [21–23]. AR-based system enables those in remote areas to receive real-time feedback on exercise. Nonetheless, studies on post-operative breast cancer patients are scarce. Although one study examined breast cancer patients post-operatively using an Xbox Kinect-based VR system, the program involved game-based motion rather than physician-prescribed exercises [13].

To our knowledge, this will be the first randomized controlled study to use an AR system for post-operative rehabilitation of breast cancer patients. Using UINCARE Home+, physicians can customize exercise programs for individual patients and receive real-time feedback on exercise accuracy and adherence. This new type of customized physical therapy program incorporating feedback may be valuable to breast cancer patients with postoperative shoulder dysfunction, especially those living in remote areas. Moreover, due to the worldwide COVID-19 pandemic, it has become difficult to provide rehabilitation therapy to patients at the hospital. In such situations, AR-based treatment is expected to be useful. In the future, AR-based physical therapy may be considered as effective tool to manage shoulder dysfunction after breast cancer surgery in addition to traditional physical therapy.

In conclusion, this study will compare the clinical efficacy of a newly developed, AR-based real-time interactive digital healthcare system with that of a brochure-based rehabilitation program, in terms of the post-operative shoulder ROM of breast cancer patients.

## Trial status

The protocol presented here was finalized in March 2020 and is version 1.0, and there is no plan to change the current protocol. The first participant recruitment began on 16 April 2020, and the study is expected to be completed at June 2022.

## Supplementary Information

**Additional file 1.**

## Data Availability

This manuscript does not contain any dataset, and no datasets were generated or analyzed during the study. The corresponding authors will have access to the final dataset of the trial.

## Abbreviations

ALND: Axillary lymph node dissection
ROM: Range of motion
VR: Virtual reality
AR: Augmented reality
CRF: Clinical research form
NRS: Numerical rating scale
QuickDASH: Shortened version of Disabilities of the Arm, Shoulder and Hand
FACT-B: Functional Assessment of Cancer Therapy-Breast
EQ-5D-5L: Five-level EuroQol five-dimensional questionnaire
